# Tunable Microgel‐Templated Porogel (MTP) Bioink for 3D Bioprinting Applications

**DOI:** 10.1002/adhm.202200027

**Published:** 2022-01-27

**Authors:** Liliang Ouyang, Jonathan P. Wojciechowski, Jiaqing Tang, Yuzhi Guo, Molly M. Stevens

**Affiliations:** ^1^ Department of Materials Department of Bioengineering Institute of Biomedical Engineering Imperial College London London SW7 2AZ UK; ^2^ Department of Mechanical Engineering Biomanufacturing and Rapid Forming Technology Key Laboratory of Beijing "Biomanufacturing and Engineering Living Systems” Innovation International Talents Base (111 Base) Tsinghua University Beijing 100084 China

**Keywords:** bioinks, bioprinting, hydrogels, microporosity, tissue engineering

## Abstract

Micropores are essential for tissue engineering to ensure adequate mass transportation for embedded cells. Despite the considerable progress made by advanced 3D bioprinting technologies, it remains challenging to engineer micropores of 100 µm or smaller in cell‐laden constructs. Here, a microgel‐templated porogel (MTP) bioink platform is reported to introduce controlled microporosity in 3D bioprinted hydrogels in the presence of living cells. Templated gelatin microgels are fabricated with varied sizes (≈10, ≈45, and ≈100 µm) and mixed with photo‐crosslinkable formulations to make composite MTP bioinks. The addition of microgels significantly enhances the shear‐thinning and self‐healing viscoelastic properties and thus the printability of bioinks with cell densities up to 1 × 10^8^ mL^−1^ in matrix. Consistent printability is achieved for a series of MTP bioinks based on different component ratios and matrix materials. After photo‐crosslinking the matrix phase, the templated microgels dissociated and diffused under physiological conditions, resulting in corresponding micropores in situ. When embedding osteoblast‐like cells in the matrix phase, the MTP bioinks support higher metabolic activity and more uniform mineral formation than bulk gel controls. The approach provides a facile strategy to engineer precise micropores in 3D printed structures to compensate for the limited resolution of current bioprinting approaches.

## Graphical abstract

A microgel‐templated porogel bioink strategy is introduced to present micropores (10–100 µm) in the cell‐laden building blocks (up to 1 × 10^8^ cells mL^−1^) for extrusion‐ and light projection‐based three‐dimensional bioprinting applications. It mediates the viscoelastic properties of a series of existing hydrogels for bioprinting and enhances embedded cell activity via better mass transport conditions.

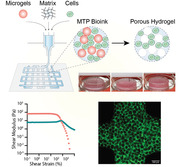

## 1 Introduction

Micropores are significantly crucial for tissue engineering scaffolds and many other 3D cell culture systems as they generate a large surface area for cell attachment and mediate mass transportation for cell growth.^[^ ^1^, ^2^, ^3^ ^]^ In the past three decades, tissue engineering has seen considerable progress with the use of porous polymeric scaffolds that are fabricated using various techniques, including freeze‐drying, salt leaching, foaming, and two‐phase emulsion.^[^ ^4^, ^5^ ^]^ A typical paradigm is that these scaffolds are seeded with cells after fabrication, as the processing conditions are often too harsh to introduce cells in situ.^[^ ^6^, ^7^, ^8^ ^]^ Building on additive manufacturing principles, 3D bioprinting has emerged as a convenient technology that allows for the free‐form manipulation of cells and biomaterials to directly engineer cell‐laden tissue constructs with high spatial resolution.^[^ ^9^, ^10^ ^]^ A commonly used model in bioprinting is a multilayered lattice structure that possesses pores between deposited filaments. These pores, usually on the scale of hundreds of microns, allow diffusion of the culture medium and facilitates access to nutrients and oxygen for cells embedded in the scaffold matrix.^[^ ^11^, ^12^ ^]^ Although more complicated geometries have been printed,^[^ ^13^, ^14^, ^15^ ^]^ incorporating finer micropores or lumen channels in large engineered tissues remains an enduring challenge.

Recent progress in the bioprinting field has advanced the engineering of pore features in hydrogel constructs. Miller et al.^[^ ^16^ ^]^ screened a series of biocompatible food dyes and used them as photoabsorbers in projection stereolithography 3D printing. The photoabsorber allowed elegant control over the printing process, and the authors could achieve complex channel networks in poly(ethylene glycol) diacrylate (PEGDA) hydrogels. Although precise intravascular topologies (most refined fin of 150 µm in thickness in a channel of 1 mm in diameter) could be printed, the pore feature is still limited to hundreds of microns.^[^ ^16^ ^]^ Feinberg et al.^[^ ^13^ ^]^ significantly advanced the extrusion‐based bioprinting technology in suspension baths. Using fine suspension microgels, they could print collagen into a well‐defined 3D construct with embedded channels down to 100 µm in diameter.^[^ ^13^ ^]^ Although smaller building blocks (either filaments, droplets, or panels) could be readily generated and used for bioassembly and bioprinting,^[^ ^17^, ^18^, ^19^ ^]^ creating a pore feature of equivalent size scale has been challenging.

Efforts have been made to engineer pores in the building block itself. For instance, Zhang et al.^[^ ^20^, ^21^ ^]^ developed an aqueous two‐phase emulsion bioink containing two immiscible aqueous phases of gelatin methacryloyl (GelMA) and poly(ethylene oxide) (PEO). After photo‐crosslinking the GelMA phase, a porous hydrogel construct was obtained by removing the PEO phase. Relying on phase separation between two immiscible hydrophilic components, the pore size and porosity were simultaneously affected by the component ratio. Moreover, it might be challenging to generalize this approach to other hydrogels due to the requirement for phase separation in aqueous solution. In another example, He et al.^[^ ^22^ ^]^ mechanically broke gelatin gel into pieces and mixed them with GelMA solution, which were then cooled to form a printable pre‐bioink. Mesoscale pores could be generated with the removal of the thermo‐sensitive gel pieces. However, the pores are in random shape and size due to the inhomogeneity of the gelatin gel pieces (size range of 100–1000 µm). In addition, porosity controlled on the mesoscale is within the printing resolution range of typical extrusion bioprinting. More recently, Heilshorn et al.^[^ ^23^ ^]^ engineered microporosity in printed acellular microgel scaffolds by using sacrificial microgels. Despite the progress, there is still a lack of facile approaches to introduce tunable micropores (especially in the range of 10–100 µm) to bioprinted cell‐laden constructs in a generalizable way.

Here, we report a tunable microgel‐templated porogel (MTP) bioink system to engineer well‐controlled pores in living building blocks. We fabricated thermosensitive gelatin microgels of varied sizes (≈10, ≈45, and ≈100 µm) and prepared MTP bioinks by mixing them with different photo‐crosslinkable formulations. As gelatin microgels would naturally dissolve and diffuse away under physiological temperature (37 °C), micropores with a size down to ≈10 µm and porosity ranging from 20% to 70% could be readily fabricated. More importantly, we found that adding our microgels in the bioinks would induce significant shear‐thinning and self‐healing properties, which are desirable rheological features for extrusion‐based bioprinting. Without the need for pre‐cooling the bioink formulation, we could print a well‐maintained 3D hydrogel construct with multiscale porosity and readily generalize the approach to other photo‐crosslinkable matrix materials. Cell experiments indicated that higher porosity in the hydrogels would induce higher metabolic activity of embedded cells. Our approach enables the engineering of highly tunable pores in cell‐laden bioinks with controlled porosity and pore dimension down to cell size‐scale. This newly developed bioink system adds to the current bioink palette and expands the bioprinting capability from the building block level.

## 2 Results and Discussion

### 2.1 Tunable Templated Microgels

**Figure 1:**
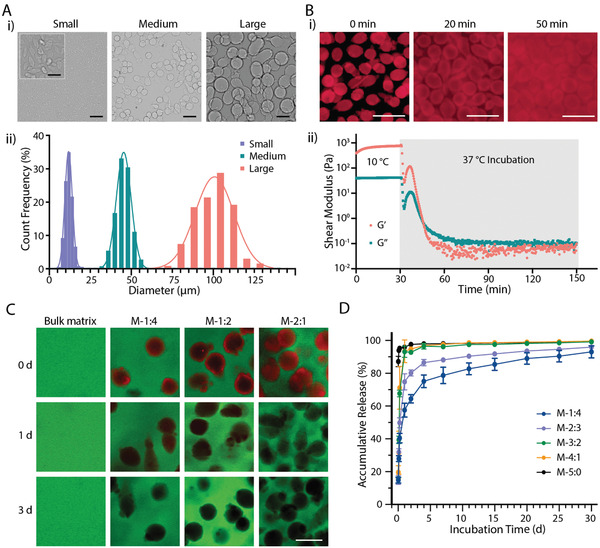
Fabrication and characterization of the templated microgels. A‐i) Representative brightfield images and ii) diameter distribution of templated microgels at different size ranges. B‐i) Fluorescence microscopy images and ii) rheological properties of microgels (size‐M) before and after 37 °C incubation. C) Fluorescence microscopy images of MTP hydrogels and D) accumulative release of gelatin from MTP hydrogels at different component ratios during 37 °C incubation. Data shown as mean ± S.D., *n* = 4. The red fluorescence in (B,C) indicates rhodamine‐labeled gelatin, and the green fluorescence in (C) indicates fluorescein‐labeled GelMA (7.5 wt%). Scale bars: A,B) 100 µm; A insert) 25 µm; and C) 50 µm.

We modified an approach from the literature^[^ ^13^ ^]^ to fabricate tunable microgels made of gelatin. Gelatin is dissolved in a water–ethanol mixture supplemented with surfactants to form an initially miscible solution under heating and stirring. When the mixture is cooled to room temperature, the gelatin phase separates from ethanol and forms spherical hydrogels due to reduced solubility in ethanol and thermal gelation in water. By screening a series of parameter configurations, we finally achieved three sets of microgels: small (size‐S) at 11.8 ± 2.1 µm, medium (size‐M) at 45.3 ± 4.6 µm, and large (size‐L) at 99.9 ± 10.6 µm (**Figure** **1**A). The geometry of the M and L microgels is highly spherical, while that of S microgels is more elongated. Nevertheless, the size distribution is highly uniform throughout the three sets, which lays the basis for forming the well‐controlled pores.

Microgels melt with time when heating at 37 °C. Fluorescence microscopy images of fluorescently labeled microgels show they fuse with each other after 20 min and lose their shape after 50 min of incubation at 37 °C (Figure 1B). Rheological time sweeps confirm the response of M‐microgels to physiological temperature. Both storage (*G*′) and loss (*G*′′) moduli decreased rapidly when heating to 37 °C, with a crossover point (*G*″ > *G*′) after ≈16 min, indicating rapid melting of the microgels (Figure 1B). Similar rheological behavior was observed in both S‐ and L‐microgels, which took ≈21 and 39 min, respectively, to reach the crossover point (Figure S1, Supporting Information). The rheological data demonstrate that the fabricated microgels retain their thermoresponsive properties.

We next added rhodamine‐labeled M‐microgels to fluorescein‐labeled GelMA solutions at different volume ratios to prepare MTP bioinks, which were then photo‐crosslinked. Initially, a higher microgel‐matrix ratio would result in denser microgels presented in the hydrogel (0 d). After incubation in PBS at 37 °C for 1 d, no obvious signal was detected in the rhodamine channel for the M‐2:1 group (M‐2:1 indicates M‐sized microgels to matrix ratio, all the following groups apply to this nomenclature), while a small rhodamine signal was detected for lower microgel ratio groups (M‐1:4 and M‐1:2) (Figure 1C). After a 3‐d incubation, a negligible fluorescence emission from the microgels was observed throughout all the groups, suggesting almost complete removal of microgels. The quantitative release study confirmed the dissociation trend of the microgels, with a higher microgel‐matrix ratio resulting in a faster rate of release (Figure 1D). When the ratio was M‐3:2 or M‐4:1, ≈95% and nearly 100% of release occurred at 1 and 7 d, respectively. The burst release is likely due to the interconnectivity of the generated pores. In comparison, for the ratio of M‐1:4 and M‐2:3, the release at 1 d was only 57.5 ± 4.2% and 74.8 ± 5.0%, respectively. Nevertheless, the release continued and reached more than 85% for both groups at 15 d (Figure 1D). These results demonstrated the successful removal of templated microgels from the matrix phase under physiological culture conditions.

### 2.2 Microgel‐Templated Porogel (MTP)

**Figure 2:**
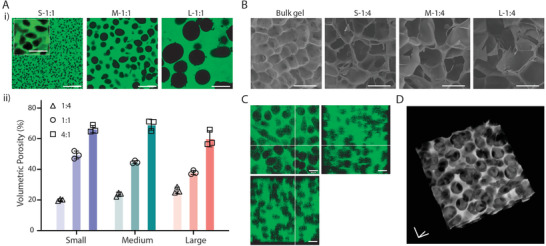
Porosity characterization of MTP hydrogels. A‐i) Representative images and ii) measured volumetric porosity of generated MTP hydrogels using templated microgels of different sizes and component ratios. Data shown as mean ± S.D., *n* = 3. B) Representative SEM images of freeze‐dried bulk gel and MTP hydrogels after 37 °C incubation for 1 d. C) Orthogonal view and D) 3D view of generated porous hydrogels with the ratio of 5:1 using Medium microgels. Scale bars: A,B) 100 µm; A insert) 25 µm; C,D in three‐axis) 50 µm.

Based on the fast dissociation and removal of microgels, microporous hydrogels of varied porosities could be fabricated. Micropores of either ≈10, ≈45, or ≈100 µm in diameter could be generated by using corresponding templated microgels (**Figure** **2**A). We further changed the microgel‐matrix ratios and measured the volumetric porosity under different configurations. A ratio of 1:4 resulted in porosities of 20.1 ± 0.7%, 23.7 ± 1.4%, and 26.3 ± 2.1% for S, M, and L‐sized microgels, respectively, without significant difference between the microgel sizes. By increasing the ratio to 1:1 and 4:1, porosities were tuned to 40–50% and 60–70%, respectively (Figure 2A; Table S1, Supporting Information). The resultant porosity values were slightly mismatched with the input volume ratios, likely due to the solution remaining in the microgel pellets after centrifugation. Nevertheless, the porosity could be fine‐tuned, and the generated pores were uniformly distributed throughout the hydrogels for all the tested groups (Figure S2, Supporting Information). All these results indicated that highly uniform and controllable porosity could be fabricated using MTP bioinks.

**Figure 3:**
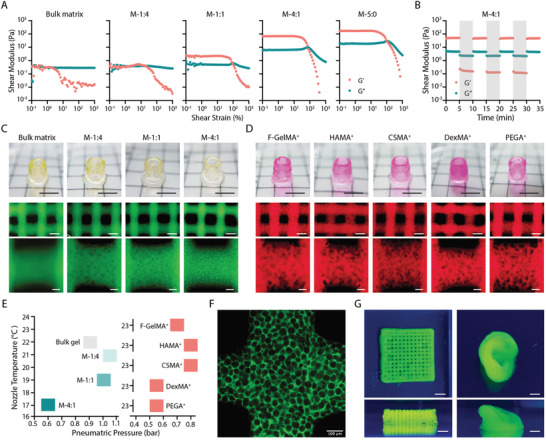
Extrusion‐based bioprinting of MTP bioinks. A) Strain sweeps of MTP bioinks at different component ratios with bulk matrix (7.5 wt% GelMA) and bulk microgels (M‐5:0) as controls. B) Time sweep of MTP bioinks (M‐4:1) under high (300%, gray shadow) and low (1%, plain) strain cycles. Frequency of 1.5 Hz and temperature of 25 °C were used in (A,B). C) Representative photos of a printed tubular structure (top panel) and microscopic images of printed lattice structure (middle and bottom panels) using MTP bioinks at different component ratios with bulk matrix (7.5 wt% GelMA) as a control. D) Representative photos of a printed tubular structure (top panel) and microscopic images of printed lattice structure (middle and bottom panels) using MTP bioinks based on M‐size microgels and different matrix materials: 10 wt% F‐GelMA^+^, 2.5 wt% HAMA^+^, 2.5 wt% CSMA^+^, 5 wt% DexMA^+^, and 5 wt% PEGA^+^. The component ratio is fixed at 1:1. E) Optimized printing parameters (nozzle temperature and pneumatic pressure) for individual bioinks presented in (C,D). F) Magnified image of a printed lattice structure using M‐4:1 bioink and G) representative photos of printed lattice and ear‐shaped structure using M‐1:1 bioink. The green fluorescence in (C,F,G) indicates fluorescein‐labeled GelMA and the red fluorescence in (D) indicates rhodamine‐labeled matrix materials. Scale bars: C,D, top panel, G) 5 mm; C,D, middle panel) 500 µm; C,D, bottom panel, F) 100 µm.

To better visualize the microstructure, we freeze‐dried the generated porous hydrogels with the bulk hydrogel as a control (Figure 2B). Under the same microgel‐matrix ratio, larger microgels resulted in larger pores after freeze‐drying. It should be noted that the observed porosity in freeze‐dried state does not necessarily represent the pore size found in the hydrated state due to phase separation during freezing.^[^ ^24^ ^]^ Nevertheless, this data confirmed the modulation of microporosity by using tunable MTP bioinks. When using a high ratio of microgels (M‐5:1), the generated pores were well connected in the matrix hydrogel, confirmed by both orthogonal (Figure 2C) and 3D views (Figure 2D; Movie S1, Supporting Information). Under these circumstances, the porous hydrogel looked like a sponge or a typical freeze‐dried porous scaffold but presented in hydrated status. It should be noted that the interconnectivity of micropores is highly dependent on the ratio of microgels presented in the bioinks. The higher the microgel to matrix ratio, the better the interconnectivity of generated pores. Under the ratio of 4:1, the estimated number of interconnecting microgels is ≈3000, ≈500, and ≈50 for S‐, M‐, and L‐sized microgels (Figure S3 and Table S1, Supporting Informtion).

### 2.3 Microgel‐Templated Porogel (MTP) Bioinks for Bioprinting

To assess the use of MTP bioinks in extrusion‐based bioprinting, we first investigated the rheological properties of bioinks under various component ratios using 7.5 wt% GelMA as the bulk matrix. GelMA at 7.5 wt% exhibits specific thermo‐gelation properties when cooling from 25 to 4 °C, with a critical gelation temperature of ≈18 °C (Figure S4A, Supporting Informtion). In comparison, the shear moduli of pure M‐size microgels do not change much during the same temperature sweep, indicating a relatively stable rheological status from 25 to 4 °C. MTP bioink of M‐1:4 acted similarly to bulk matrix, with a slightly higher initial storage modulus (in the temperature range of 20‐25 °C), while the M‐4:1 bioink behaved more like the pure microgels without a sharp change of the shear moduli. M‐1:1 bioink maintained a shift in storage modulus around 16 °C, indicating slight thermo‐sensitivity (Figure S4A, Supporting Informtion).

The bulk matrix behaved like a viscous solution at 25 °C, retaining low shear moduli during the strain sweep from 0.01 to 1000% (**Figure** **3**A). However, when the temperature was set at 15 °C (gelation has initiated), *G*′ significantly increased to ≈1400 Pa at the low strain region and dramatically decreased to ≈40 Pa at a strain of 1000% (Figure S4B, Supporting Informtion). Thus, to enhance the viscosity and printability of GelMA, a pre‐cooling process has been used in the literature,^[^ ^22^, ^25^ ^]^ which might cause over gelation and nonuniform filaments.^[^ ^18^, ^26^ ^]^ Our data demonstrated that the addition of gelatin microgels would significantly induce shear‐thinning and self‐healing properties at 25 °C (Figure 3A), which is favourable for extrusion‐based bioprinting.^[^ ^27^ ^]^ The M‐1:1 bioink displayed a linear viscoelastic region (LVR) from 0.01 to ≈30% strain – *G*′ kept at ≈2 Pa and then decreased to ≈0.01 Pa at the strain of 1000%. The M‐4:1 and pure microgel bioinks presented much higher *G*′ (≈70 and 3170 Pa, respectively) at LVR (Figure 3A). Under high (300%) and low (1%) strain cyclic sweeps, *G*′ and *G*′′ of M‐4:1 bioink showed complete recovery of *G*′ and *G*′′, indicating a fast sol–gel transition (Figure 3B). Highley et al. has demonstrated the use of photo‐crosslinked microgels as an innovative form of bioink, which presented a significant shear‐thinning and self‐healing property.^[^ ^28^ ^]^ Confirming this conclusion, our data further indicated that microgel‐matrix mixtures at certain ratio could also deserve similar rheological properties, in favor of extrusion‐based 3D bioprinting.

We then optimized the 3D printing of MTP bioinks (Figure 3C,D). All the tested MTP bioinks based on GelMA (M‐1:4, M‐1:1, M‐4:1) could be printed into a standard tubular structure with excellent integrity as compared with the GelMA control (Figure 3C). The typical lattice structure confirms the consistent printability in terms of overall geometry. Furthermore, the microscopic images indicated increasing porosity with the increase of the component ratio (Figure 3C). For bioinks with a higher microgel ratio (e.g., M‐4:1), slightly lower nozzle temperature and pneumatic pressure were used to yield similar printability (Figure 3E). This is likely due to the change of the thermo‐gelation property in the presence of microgels. After incubating at 37 °C for one week, all the printed constructs maintained the macro geometry and microporosity (Figure S5, Supporting Informtion), indicating the stability of the MTP bioink and the printed structure. To further demonstrate the generalizability of our approach, we prepared MTP bioinks using a series of complementary network formulations^[^ ^29^ ^]^ as the corresponding matrix phase. These complementary network formulations include those based on cold fish GelMA (F‐GelMA), methacrylated hyaluronic acid (HAMA), methacrylated chondroitin sulphate (CSMA), methacrylated dextran(DexMA), and 8‐arm polyethylene glycol acrylate (PEGA, *M_n_* = 40 kDa), which are all supplemented with the same amount of soluble gelatin (5 wt%) as in the literature.^[^ ^29^ ^]^ All the tested groups showed excellent printability with both tubular and lattice structures (Figure 3D). The printing temperature for all the groups was fixed at 23 °C, while the pneumatic pressure was 0.5–0.8 bar (Figure 3E). These data further demonstrated the generalizability of our approach to expand the bioink library with excellent printability and microporosity features (Figure 3F,G).

**Figure 4:**
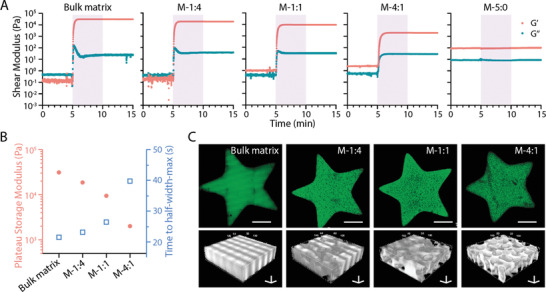
Projection‐based printing of MTP bioinks. A) Photorheological properties of MTP bioink at varied component ratios with bulk matrix (20 wt% fish GelMA) and bulk microgels as controls. The shaded area indicates UV exposure (10 mW cm^−2^). B) Effect of component ratios on the maximum storage modulus and time to half‐width‐max during photo‐crosslinking. C) Fluorescent images and 3D views of printed star‐shaped structures. Green fluorescence indicates fluorescein‐labeled fish GelMA (20 wt%). Scale bars: C, top panel) 500 µm; C, bottom panel, three‐axis) 50 µm.

We also applied the MTP bioinks to stereolithographic patterning techniques. To enable the flow of the bioink resin, here we chose to use cold fish GelMA as the matrix phase considering its low viscosity of up to 20 wt% throughout a wide temperature range (4–37 °C) (Figure S6, Supporting Informtion). When exposed to UV light, 20 wt% F‐GelMA was immediately crosslinked (**Figure** **4**A) with the time to half‐width‐max of ≈22 s and a plateau storage modulus of 3 × 10^4^ Pa when fit to an empirical Gompertz function (Figure 4B). In comparison, the plain microgel formulation presented a higher initial *G*′ and *G*′′, which was unaffected by UV exposure. Interestingly, the MTP bioinks with different ratios were all photo‐crosslinked in the presence of UV light. Generally, the higher microgel ratio would result in longer time to half‐width‐max and lower *G*′ at the plateau stage (Figure 4B). The addition of microgels to the photo‐crosslinkable phase likely affects the diffusion of the GelMA and thus slowed down the crosslinking kinetics. At the same time, the decreased ratio of crosslinked polymer was likely to induce a lower shear modulus. Nevertheless, adding up to 4:1 ratio of microgels could still induce a typical photo‐crosslinking response, which took ≈40 s to reach half‐width‐max and resulted in a *G*′ plateau of ≈2 × 10^3^ Pa. Next, we loaded the inks on a masked steolithographic (MSLA) 3D printer and patterned a star‐shaped structure. M‐1:4 and M‐1:1 bioinks show similar structures to the bulk matrix bioink, while increasing the microgels in the formulation (M‐4:1 bioink) seemed to affect the photo‐crosslinking process and thus slightly compromised the fidelity (Figure 4C). Nevertheless, all the groups were successfully patterned into porous constructs (Figure 4C). These early‐stage data demonstrated that the microgels could survive in the light projection process, which laid the basis for printing microporous 3D constructs in the future.

### 2.4 Cell Printing with MTP Bioinks

**Figure 5:**
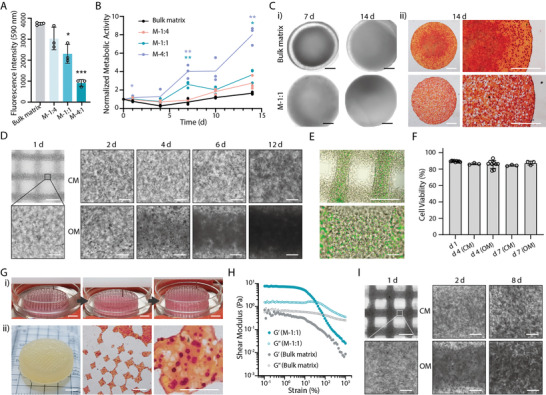
Bone tissue engineering. A) Initial fluorescent intensity (day 0) and B) normalized metabolic activity of cell‐laden MTP hydrogels during 14‐d culture using the alamarBlue assay. C‐i) Microscopic images of cell‐laden MTP hydrogels during culture and ii) alizarin Red S staining on day 14. A bulk matrix of 7.5 wt% GelMA was used as a control. D) Representative microscopic images of printed lattice structure using M‐1:1 bioinks during culture using culture medium (CM) or osteogenic medium (OM). E) LIVE/DEAD staining of printed lattice structure on day 1 and F) quantitative cell viability during culture. Data shown as mean ± S.D., *n* ≥ 3. G‐i) 3D bioprinting of centimetre sized porous constructs and ii) representative photo and alizarin Red S staining images of the printed constructs after culturing for 14 d. Saos‐2 cells with density of 5 × 10^6^ cells mL^−1^ in the matrix were used in (A–G). H) Rheological properties of bulk matrix and MTP bioink containing super high density of Saos‐2 cells (1 × 10^8^ mL^−1^). I) Representative microscopic images of printed lattice structure (M‐1:1 bioink with 1 × 10^8^ cells mL^−1^ in matrix phase). Two‐tailed *t*‐test, *p < 0.05, **p < 0.01, ***p < 0.001 (data shown as mean ± S.D., *n* = 3). Scale bars: C,E; D,I, top‐left; G‐ii, middle and right)1 mm; D,I, except top‐left) 100 µm; G‐i; G‐ii, left) 5 mm.

To determine the effect of microgels and micropores on the cell viability, we embedded osteoblast‐like Saos‐2 cells in the matrix phase and performed an alamarBlue assay. Initially, an increase of the M‐size microgels in the formulation would induce lower fluorescence intensity, which correlated to the number of cells present in matrix phase (**Figure** **5**A). To better evaluate the metabolic activity of cells during a two‐week culture, we normalized the data to day 0. The results indicated that a higher ratio of microgel‐matrix would result in higher metabolic activity and a faster proliferation rate (Figure 5B). This was also true when using S‐ or L‐size microgels (Figure S7, Supporting Informtion). These results confirmed that incorporating micropores could enhance the cell activity, which is likely due to a better mass transport condition.

When culturing the cell‐laden constructs in an osteogenic medium (OM), hydroxylapatite would be produced by the osteoblast‐like cells. After culturing for 7 or 14 d, the mineral mainly accumulated in the central area of the construct in the bulk hydrogel, while uniform opaque mineral was found in M‐1:1 hydrogel (Figure 5C). The alizarin red S staining confirmed this observation that more calcium was retained in the center of the bulk hydrogel, while calcium was uniformly distributed throughout the M‐1:1 hydrogel (Figure 5C). Thus, we applied the M‐1:1 bioink to the printing of bone‐like tissue constructs.

After printing, the cell‐laden lattice constructs were cultured in culture medium (CM) for 1 d and then changed with fresh CM or OM every two days. Cells were distributed evenly in the constructs, and micropores could be observed surrounding the cells (Figure 5D; Figure S8A, Supporting Informtion). When maintaining in CM, cells kept proliferating, with more cells observed in the constructs until 12 d. In comparison, when culturing in OM, the constructs turned opaque, and minerals accumulated with time. The embedded cells maintained uniform living cells after 1 d (Figure 5E) and high cell viability (>85%) throughout one‐week culture in CM or OM (Figure 5F). These results demonstrated the excellent biocompatibility of MTP bioinks for cell printing. We further fabricated a centimeter‐sized porous construct and cultured it for 14 d in OM. Every layer was printed smoothly with the deposition of standard filaments without defects. After 14 d, the large construct turned opaque evenly, and alizarin red S staining indicated uniform calcium deposition (Figure 5G).

To meet tissue engineering requirements for cell density of native tissues, we further demonstrated the feasibility of printing bioinks with high cell densities. We prepared a GelMA matrix phase containing 1 × 10^8^ Saos‐2 cells mL^−1^ and mixed it with microgels to prepare M‐1:1 bioink. The bulk matrix bioink presented a modulus below 1 Pa throughout the strain range, while the M‐1:1 bioink presented a much higher storage modulus at LVR and significant shear‐thinning properties (Figure 5H). This was also true for the bioinks with other component ratios (Figure S9A, Supporting Informtion). These data suggested that the use of a super high concentration of cells in the bioink would retain the favorable rheological properties of MTP bioink for extrusion‐based bioprinting. Indeed, the M‐1:1 bioink with dense cells could be printed into a standard 3D lattice structure, which was visualized with much higher cell density at the early stage (1 d) (Figure 5I; Figure S8B, Supporting Informtion). Similar to the results with lower cell density, cells proliferated with time and produced calcium when culturing in OM (Figure 5I; Figure S8B, Supporting Informtion), while maintaining a considerable cell viability throughout one‐week culture (>75%) (Figure S9B, Supporting Informtion). Our data demonstrated the feasibility of having super high cell density in the MTP bioinks to fabricate in vitro tissues.

## 3 Conclusion

In summary, we developed a microgel‐templated porogel bioink system for 3D bioprinting in order to mediate the microporosity in fabricated cell‐laden constructs. This approach allows for the engineering of micropores in the living building block level with well‐defined porosity (20‐70%) and pore size down to ≈10 µm. The concept of porous bioink adds to the innovative forms of bioinks and offers previously unidentified capability of bioprinting in terms of porosity engineering. Compared to the typical methods (e.g., freeze‐drying) to prepare porous scaffolds, our approach enables incorporating living cells in situ for biomimetic 3D culture. The addition of microgels in the bioinks induces favorable shear‐thinning and self‐healing properties for extrusion‐based bioprinting. Various existing bioink formulations could be readily adapted to composite MTP bioinks while maintaining consistent printability. Using a suitable matrix phase, the MTP bioinks could also be applied to the projection‐based printing process. Moreover, MTP bioinks support higher metabolic activity of encapsulated cells than their bulk matrix counterpart does. Together, our work opens a new avenue to develop cell‐laden bioinks for cell niche engineering and tissue engineering with better mass transportation.

## 4 Experimental Section

### Materials Synthesis

Photo‐crosslinkable matrix materials were synthesized by introducing methacrylate or acrylate functional groups to the polymer backbones using previously reported protocols.^[^ ^29^ ^]^ The modification degrees of GelMA, HAMA, CSMA, DexMA, and PEGA were ≈80%, ≈25%, ≈65%, ≈10%, and >95%, respectively.^[^ ^29^ ^]^ GelMA was synthesized from porcine gelatin (Type A, ≈300 g bloom, G1890 Sigma Aldrich) and unless otherwise stated, all gelatin represents this source. Fish GelMA (F‐GelMA) was synthesized from fish skin gelatin (G7041 Sigma Aldrich) according to literature^[^ ^30^ ^]^ and the degree of functionalization is 79 ± 0.6% (Figure S10, Supporting Informtion). The detailed synthesis and characterization of F‐GelMA can be found in the Supporting Information. Fluorescein‐ and rhodamine‐conjugated gelatin or GelMA were synthesized by reacting with NHS‐Fluorescein and NHS‐Rhodamine (Thermo Fisher Scientific), respectively. Gelatin and GelMA were fully dissolved in phosphate buffer (pH ≈ 8.1) at 50 °C at a concentration of 10 wt%, followed by the addition of NHS‐Fluorescein or NHS‐Rhodamine (30 mg per 1 g of gelatin). After 3–8 h reaction at 50 °C in the dark, the mixture was dialyzed against pure water at 40 °C for 1 week. After freeze‐drying, all the synthesized materials were stored in −20 °C freezer until use.

### Fabrication of Templated Microgels

The gelatin templated microgels were fabricated using a method adapted from literature.^[^ ^13^ ^]^ Gelatin (Sigma, G1890) was first dissolved in warm water at a concentration of 4 wt%, followed by adding a certain amount of ethanol. While stirring and heating at 50 °C, the mixture was added with certain amounts of pluronic F127 (Sigma) and gum arabic (Sigma). For small (size‐S) microgel fabrication, ethanol was added at 4:5 volume ratio of water, while PF127 and gum arabic were added at 0.125‐ and 0.05‐times mass of gelatin, respectively. For medium (size‐M) and large (size‐L) microgel fabrication, the fractions (of ethanol, PF127, and gum arabic) were (1, 0.125, and 0.05) and (1, 0.25, and 0.1), respectively. After fully dissolving, the reaction was placed at room temperature (24–25 °C) while stirring at 400 RPM overnight. The microgel‐containing solution was centrifuged at 300 g for 5 min and the supernatant was discarded. The microgel pellets were washed with phosphate buffer saline (PBS), followed by centrifugation at 1000 × *g* and 4 °C for 5 min. The washing step was repeated three times to minimize the retention of ethanol and surfactants. The collected microgels were finally resuspended in PBS and stored at 4 °C fridge until use. For the cell culture study, the microgels were additionally washed with culture medium three times. To visualize the microgels and detect the dissolved compound, fluorophore‐labeled gelatin was used to fabricate the microgels.

### Preparation of MTP Bioinks

Empty tubes were weighted first and loaded with microgel suspension, followed by centrifugation at 2000 × *g* and 4 °C for 10 min. After discarding the supernatant carefully, the tubes containing microgels were weighed again and the mass of microgels was determined by subtracting the original tube mass. To prepare MTP bioink, the bulk microgels were then resuspended with bulk matrix (matrix hydrogel precursor solution) at a certain component ratio using positive displacement pipettes. The component ratio of M‐1:4 indicates that 1 mg size‐M microgels were mixed with 4 µL bulk matrix. For photo‐crosslinkable matrix materials, 4 ×10^−3^ m photoinitiator (lithium phenyl‐2,4,6‐trimethylbenzoylphosphinate, LAP) was included in the bulk matrix solution for extrusion‐based bioprinting, while 34 ×10^−3^ m LAP and 0.5 ×10^−3^ m tartrazine were included for stereolithography printing. The MTP bioinks were prepared fresh, and the process was carried out at room temperature (24‐25 °C) to minimize melting of microgels and solidification of matrix phase. 

### Rheological Characterization

Rheological measurements of the hydrogels were recorded using an Anton Paar MCR302 rheometer fitted with a 25 mm stainless steel parallel plate (PP25) and Peltier temperature‐controlled hood (P‐PTD200/80/I). To determine the thermal responsivity of different formulations, oscillatory temperature sweeps were carried out with temperature ramp change at a rate of 5 °C min^−1^, while shear strain and frequency were fixed at 1% and 1.5 Hz, respectively. In addition, a sharp temperature change from 10 to 37 °C was applied to the oscillatory time sweeps to demonstrate the successful melting of microgels under physiological temperature. Oscillatory strain and frequency sweeps were performed with a fixed frequency (1.5 Hz) and strain (1%), respectively. To determine the photo‐crosslinking kinetics, time sweeps at 25 °C were performed, during which in situ light irradiation (365 nm, 10 mW cm^−2^, OmniCure S1500) was applied for 5 min. Unless otherwise stated, constant values of strain, frequency and temperature were 1%, 1.5 Hz, and 25 °C, respectively.

### Microscopy

To visualize and distinguish the microgel and matrix phases, fluorophore‐labeled polymers were used to prepare individual phases. All optical microscope images were taken using a widefield fluorescence microscope (Olympus BX51) or a confocal laser scanning microscope (Leica SP5). The size of microgels and porosity of MTP hydrogels were measured using the Analyze Particles function in ImageJ. To observe the microstructure of different hydrogels, hydrogels were prepared and incubated at 37 °C for 1 d, followed by freeze‐drying. The freeze‐dried samples were then sputter‐coated with gold and imaged with a Zeiss Auriga scanning electron microscope (SEM) system at 5 kV.

### Release of Gelatin

To determine the removal of gelatin microgels from MTP hydrogels, fluorescein‐labeled microgels were used and the release of gelatin was detected with time. In a typical experiment, 40 µL MTP bioinks at a certain component ratio were cast in a disposable truncated syringe (1 mL, BD), followed by UV treatment (365 nm, 10 mW cm^−2^, 5 min). The generated hydrogels were placed in separate Eppendorf tubes filled with 1 mL of PBS for incubation at 37 °C. At given time points, 500 µL supernatant was collected and 500 µL fresh PBS was added back to the hydrogel. After the final collection, the remaining mixture was homogenized for 10 min at 22 Hz using a TissueLyser II (QIAGEN). The amount of gelatin was measured by the fluorescence intensity at 525 nm (excitation at 490 nm) using a plate reader (SpectraMax M5). The accumulative percentage release with time was calculated by normalizing to the total amount of gelatin.

### Extrusion‐Based 3D Bioprinting

Bioinks were made fresh and loaded to 50 cc cartridge, which was then placed in the printer (3D Bioplotter, EnvisionTec) nozzle set at a certain temperature. The nozzle temperature was determined when the bioinks could hang a smooth filament at the nozzle during manual extrusion. The pneumatic pressure was determined when the bioinks was extruded at a volume equal to an ≈1 mm diameter spheroid during 1 s purge. Printing speed was set at 2.5 mm s^−1^ for tubular and thin lattice structures. For centimeter‐sized structure printing, printing speed was set at 10 mm s^−1^ with a higher pneumatic pressure accordingly. Unless otherwise stated, 25‐gauge needle (inner diameter of 260 µm) was used for all the tested groups. Due to the presence of a continuous matrix phase and the smaller size of microgels than the needle, the printing process was smoothly conducted without observing undesired aggregating or jamming of microgels within the bioinks. After printing, the constructs were treated with UV light (10 mW cm^−2^, 2.5 min in the air plus 2.5 min in photoinitiator solution), followed by 37 °C incubation.

### Light Projection‐Based Printing

A masked stereolithographic 3D printer (Prusa SL1) was used to pattern customized 2D structures. In a typical experiment, a 20 wt% fish‐GelMA containing 34 × 10^−3^ m LAP and 0.5 × 10^−3^ m tartrazine was used as the bulk matrix bioink. M‐size microgels were mixed with bulk matrix at different ratios to prepare MTP bioinks. A star‐shaped model was printed out via a photomask with UV exposure time of 5 min.

### Cell Culture and Cell Printing

Human bone osteosarcoma cells (Saos‐2, American Type Culture Collection) were cultured in *α*‐modified Eagle′s medium (*α*‐MEM) supplemented with 10 vol% FBS and 1 vol% P/S at 37 °C, 5% CO_2_. Before cell printing, cells were trypsin‐treated and collected as pellets. The single cell suspension was then mixed with bulk matrix solution at a density of 7.5 × 10^6^ or 1 × 10^8^ cells mL^−1^. Fresh microgels were washed with culture medium three times before mixing with cell‐laden matrix solution to prepare MTP bioink. After printing and photo‐crosslinking the constructs, culture medium was added and changed every two days for culturing at 37 °C, 5% CO_2_.

### Cell Activity Characterization

An alamarBlue assay (Thermo Fisher Scientific) was used to determine the metabolic activity of cells embedded in hydrogels. Briefly, alamarBlue reagent was diluted with culture medium (1 in 10 dilution) to prepare the working solution. At defined time points, cell‐laden constructs were incubated with the working solution for 3 h, followed by the fluorescence reading (emission wavelength of 590 nm, excitation wavelength of 560 nm) of the supernatant using a multimode plate reader (EnVision). To determine cell viability, LIVE/DEAD staining was conducted by immersing the cell‐laden constructs into calcein‐AM/ethidium homodimer‐1 (Invitrogen) working solution (each at 1 × 10^−6^ m) for 20 min. To assess calcium production, bone‐like tissue constructs were fixed with 4% paraformaldehyde for 2 h and washed 70 vol% ethanol for paraffin embedding. Sections (10 µm) on SuperFrost Plus slides (Thermo Scientific, UK) were stained with Alizarin Red S (2 wt%, pH 4.2) for 2 min. The stained slides were dehydrated in 70 and 100 vol.% ethanol sequentially, followed by drying and mounting in Histomount (National Diagnostics). A widefield fluorescence microscope (Olympus BX51) or a confocal laser scanning microscope (Leica SP5) was used to obtain images.

### Statistical Analysis

All statistical analyses were performed using GraphPad Prism X9. Unless otherwise noted, all data were presented as mean ± SD, and all statistical comparisons were made using a two‐tailed *t*‐test with Welch's correction.

## Conflict of Interest

The authors declare no conflict of interest.

## Author Contributions

L.O. conceived the study and performed the experiments. M.M.S. supervised the study. L.O. wrote the manuscript with input from other authors. J.P.W. contributed to the materials synthesis and stereolithography printing. J.T. contributed to the SEM imaging. Y.G. contributed to the data processing.

## Supporting information

Supporting Information

Supporting Information

## Data Availability

All data needed to evaluate the conclusions in the paper are present in the paper and/or the Supplementary Materials. Raw research data is available at DOI: 10.5281/zenodo.5841653.
