# Supplementary material for: Tunable Microgel-Templated Porogel (MTP) Bioink for 3D Bioprinting Applications
Source: Adv Healthc Mater. Author manuscript; Available in PMC 2024 Jan 6. (PMC7615490; doi:10.1002/adhm.202200027)
Supplement: Supplementary Materials [file EMS175892-supplement-Supplementary_Materials.pdf]

ADVANCED HEALTHCARE MATERIALS

Supporting Information

for *Adv. Healthcare Mater.*, DOI 10.1002/adhm.202200027

Tunable Microgel-Templated Porogel (MTP) Bioink for 3D Bioprinting Applications

Liliang Ouyang, Jonathan P. Wojciechowski, Jiaqing Tang, Yuzhi Guo and Molly M. Stevens**

WILEY-VCH

ADVANCED HEALTHCARE MATERIALS

Supporting Information

for *Adv. Healthcare Mater.*, DOI: 10.1002/adhm.202200027

Tunable Microgel-Templated Porogel (MTP) Bioink for 3D Bioprinting Applications

Liliang Ouyang, Jonathan P. Wojciechowski, Jiaqing Tang, Yuzhi Guo, Molly M. Stevens

Supplemental Information

Tunable Microgel-Templated Porogel (MTP) Bioink for 3D Bioprinting Applications

Liliang Ouyang, Jonathan P. Wojciechowski, Jiaqing Tang, Yuzhi Guo, Molly M. Stevens

Fish GelMA Synthesis

Fish skin derived GelMA (F-GelMA) was synthesised following a literature procedure [1]. In a typical experiment, 20 g of fish skin derived gelatin (Sigma Aldrich, product number: G7041-500G, lot number: SLBX7992) was weighed into a 500 mL round bottom flask and dissolved at room temperature in 200 mL of 0.25 M carbonate buffer (pH = 9). The mixture was stirred vigorously (500 RPM or greater) then methacrylic anhydride (0.05 mL/g) was added dropwise. The reaction mixture was capped with a glass stopper and allowed to stir at room temperature for 5 hours. The pH was then adjusted to 7 using a solution of 1 M hydrochloric acid and transferred to dialysis tubing (SpectraPOR, standard RC, 6-8 kDa MWCO). The F-GelMA was dialysed against milliQ H¬2O (5 L) and protected from light by covering with aluminium foil. The milliQ H2O was changed 6 times over 2 days. The dialysed F-GelMA solution was transferred to 50 mL falcon tubes and lyophilised to give 15.4473 g of F-GelMA as a spongy solid. To determine the degree of functionalisation, the F-GelMA was measured using a fluoraldehyde assay and ^{1}H NMR in D_2O using sodium 3-trimethylsilyl-propionate-2,2,3,3-d_4 (TMSP) as an internal standard.

Fluoraldehyde Assay

The fluoraldehyde reagent (Thermo Scientific. REF: 26025. LOT: UB276239) was aliquoted into a 15 mL falcon tube and warmed to room temperature. Stock solutions of fish skin derived gelatin (1, 0.5, 0.1 and 0.02 mg/mL) and fish-GelMA samples at 1 mg/mL were prepared in 1xPBS. A 300 μL of each sample was mixed with 600 μL of the fluoraldehyde reagent and then 3x250 μL of each mixture was transferred to a 96-well fluorescence plate. The plate was read

using excitation wavelength = 350 nm and emission wavelength = 450 nm). A calibration curve was calculated from the standards. The degree of functionalisation (%) of the GelMA was determined using the equation (1 - X)/1*100, where X = determined concentration of GelMA. The determined concentration was 79±0.7%.

^{1}H NMR

^{1}H NMR spectra was recorded on a JEOL ECZ400S NMR spectrometer operating on a frequency of 400 MHz. NMR spectra were recorded at 298 K in D_2O, with the chemical shifts being referenced to sodium 3-trimethylsilyl-propionate-2,2,3,3-d_4 (TMSP), $\delta = 0$ ppm at a concentration of 1 mg/mL. The degree of modification of F-GelMA was measured following the methods described by Claaßen *et al*. [2] The degree of modification was taken as the average of three samples. Average number of methacryloyl groups = 0.292±0.002 mmol/g. Average number of methacrylamide groups = 0.275±0.004 mmol/g. Average number of methacrylate groups = 0.0171±0.004 mmol/g.

References

1. H. Shirahama, B. H. Lee, L. P. Tan, N. J. Cho (2016). Precise Tuning of Facile One-Pot Gelatin Methacryloyl (GelMA) Synthesis. Scientific reports 6, 31036.
2. C. Claassen, M. H. Claassen, V. Truffault, L. Sewald, G. E. M. Tovar, K. Borchers, A. Southan (2018). Quantification of Substitution of Gelatin Methacryloyl: Best Practice and Current Pitfalls. Biomacromolecules 19, 42-52.

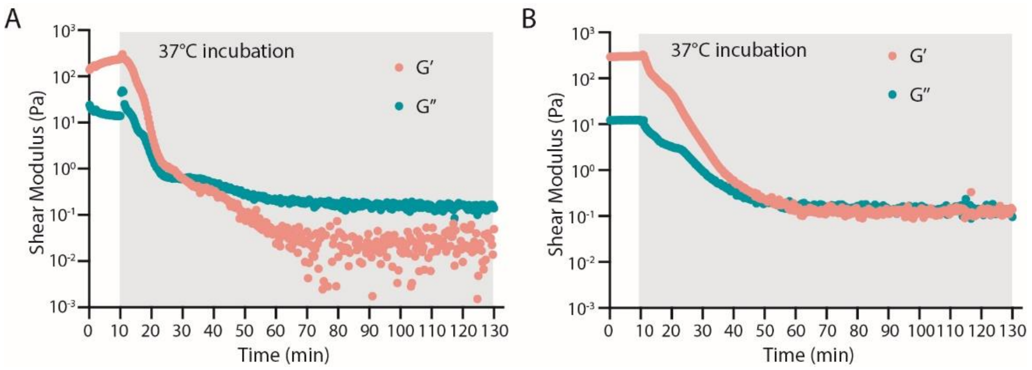

Figure S1. Thermo-rheological properties of gelatin microgels of (A) small and (B) large sizes. A frequency of 1.5 Hz and strain of 1 % was used. The temperature was set at 10 °C in the first 10 minutes.

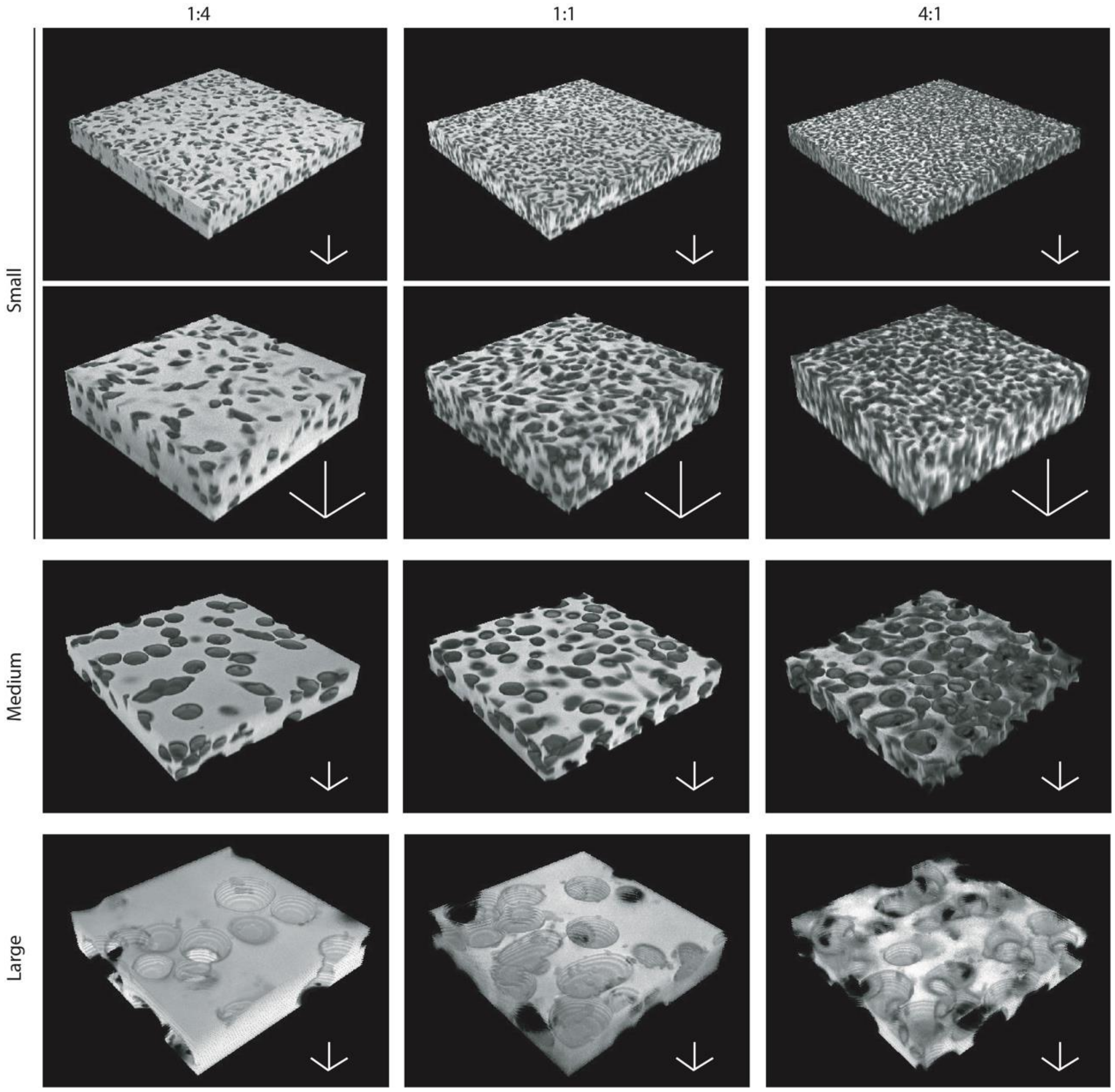

Figure S2. 3D view of porous hydrogels fabricated from MTP bioinks with different pore sizes and porosity. Scale bars in in three-axis: 50 μm.

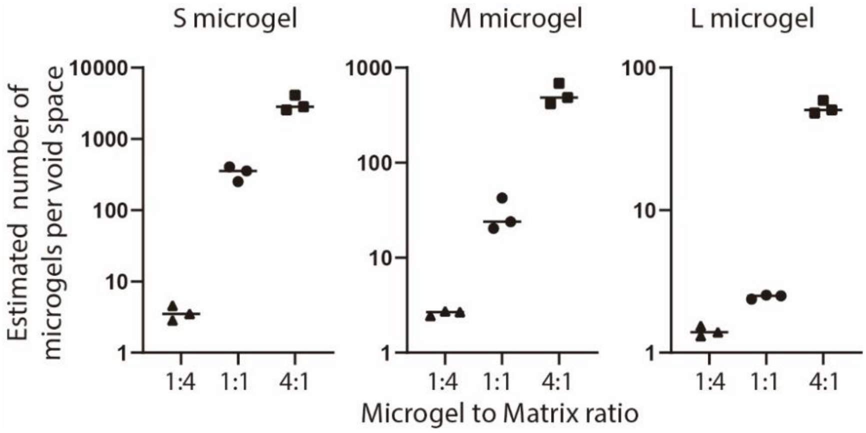

Figure S3. Estimated number of microgels per void space in the porous hydrogels obtained from different parameters. Data show as mean ± S.D., n = 3.

Table S1. Void analysis of porous hydrogels obtained under different parameters (triplicates for each condition). The volume of single microgel is determined by the estimated diameter in Figure 1. Porosity is calculated by dividing total volume of space by void volume, both are obtained from confocal imaging analysis. The estimated number of microgels per void represents the extent of contacting of microgels or interconnectivity of spherical pores.

Samples	Total volume (μm^3)	Void volume (μ m^3)	Void number	Volume per void (μm^3)	Volume of single microgel (μm^3)	Porosity (%)	Estimated number of microgels per void
S-1:4	25749882	5218399	1318	3959.3	861.1	20.27	4.6
	15525664	2993134	994	3011.2	861.1	19.28	3.5
	2394437	495369.5	202	2452.3	861.1	20.69	2.8
S-1:1	37867473	17772511	82	216737.9	861.1	46.93	251.7
	39916771	19629722	56	350530.7	861.1	49.18	407.1
	34838075	18138940	59	307439.7	861.1	52.07	357.0
S-4:1	31808677	21940668	9	2437852	861.1	68.98	2831.0
	27264580	17624163	8	2203020	861.1	64.64	2558.3
	27264580	17771834	5	3554367	861.1	65.18	4127.5
M-1:4	62102656	15436340	116	133071.9	48774.6	24.86	2.7
	62102656	13723900	115	119338.3	48774.6	22.10	2.4
	60587957	14553989	111	131117	48774.6	24.02	2.7
M-1:1	60587957	26864489	23	1168021	48774.6	44.34	23.9
	58449558	25744269	26	990164.2	48774.6	44.05	20.3
	59073258	27047921	13	2080609	48774.6	45.79	42.7
M-4:1	46955666	33442540	1	33442540	48774.6	71.22	685.7
	36352774	23635832	1	23635832	48774.6	65.08	484.6
	28779279	20431930	1	20431930	48774.6	71.00	418.9
L-1:4	63617354	16165798	20	808289.9	523934.4	25.41	1.5
	63617354	15779953	23	686084.9	523934.4	24.80	1.3
	63617354	18273460	25	730938.4	523934.4	28.72	1.4
L-1:1	63617354	23948764	18	1330487	523934.4	37.65	2.5
	59875157	23626373	18	1312576	523934.4	39.46	2.5
	60587957	22370422	18	1242801	523934.4	36.92	2.4
L-4:1	46955666	30957278	1	30957278	523934.4	65.93	59.1
	46955666	26483308	1	26483308	523934.4	56.40	50.5
	43926269	25178974	1	25178974	523934.4	57.32	48.1

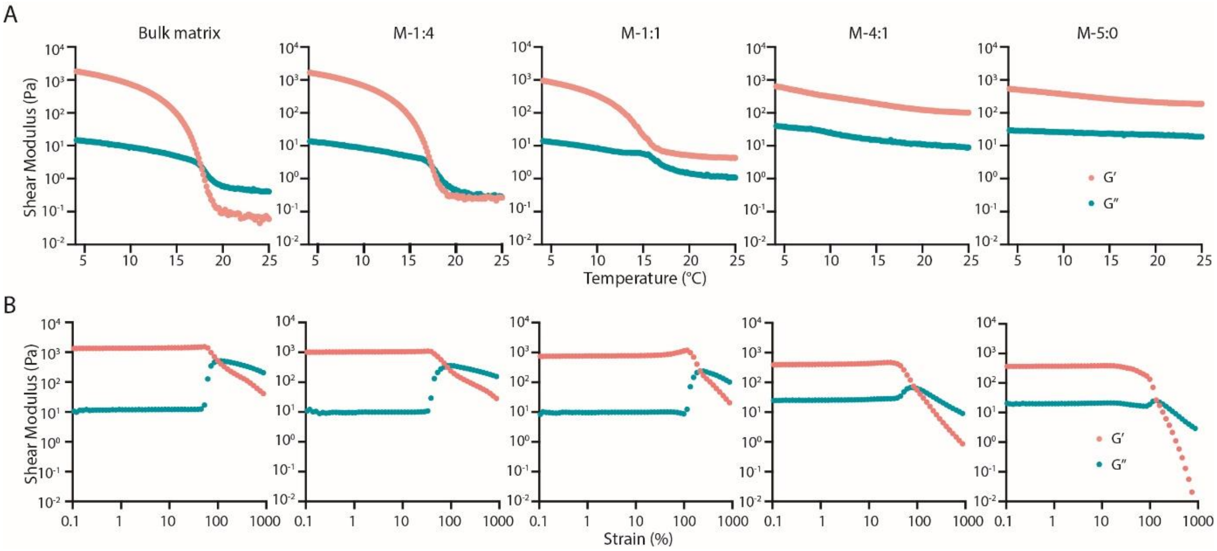

Figure S4. Rheological properties of different MTP bioinks (with the bulk matrix of 7.5 wt% GelMA and bulk microgels as controls) under different measurement configurations: (A) temperature sweeps (cooling) at a strain of 1% and frequency of 1.5 Hz; (B) strain sweeps at a temperature of 15 °C with a fixed frequency of 1.5 Hz.

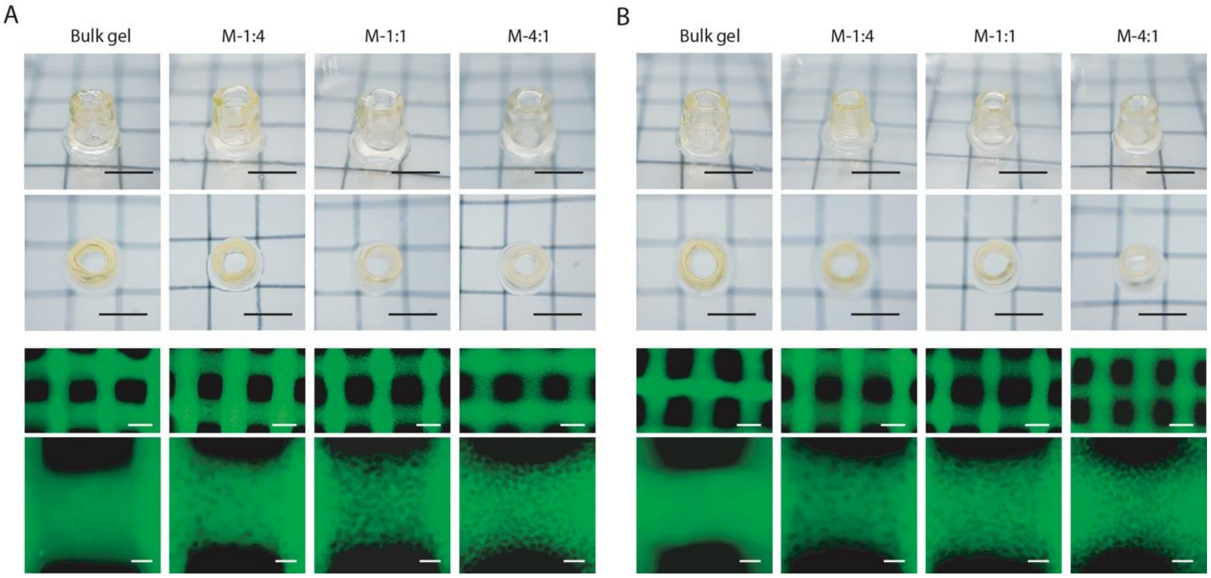

Figure S5. Representative images of printed tubular and lattice structures on (A) day 1 and (B) day 7 using different MTP bioinks with the bulk matrix as a control. Scale bar: 5 mm (photographies), 500 µm and 100 µm (fluorescence images).

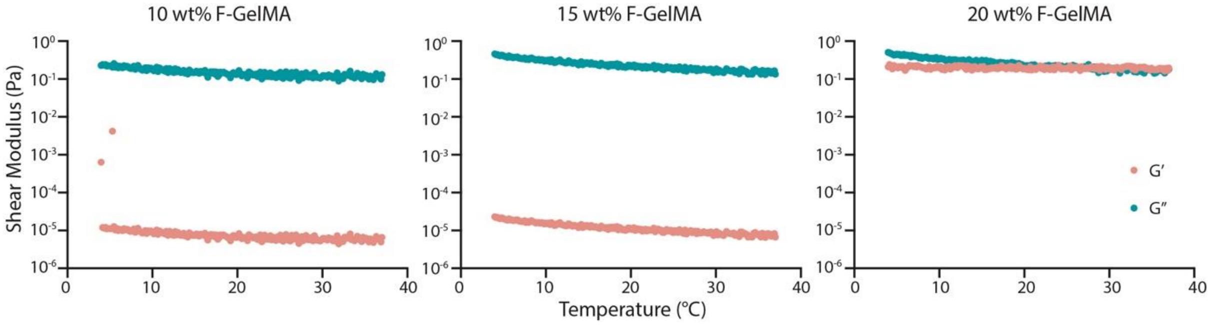

Figure S6. Temperature sweeps (cooling) of F-GelMA at different concentrations at a strain of 1% and frequency of 1.5 Hz.

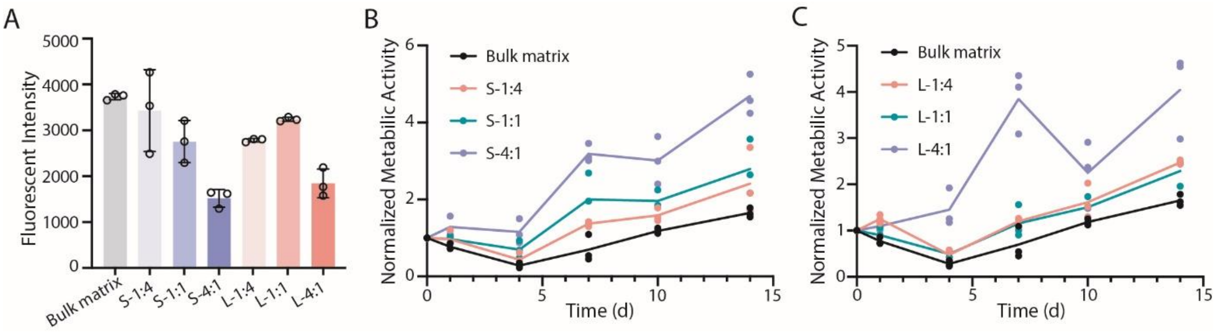

Figure S7. (A) Initial fluorescence intensity (day 0) and (B-C) normalized metabolic activity of cell-laden hydrogels (7.5 wt% GelMA) during 14-day culture using the alamarBlue^{TM} assay.

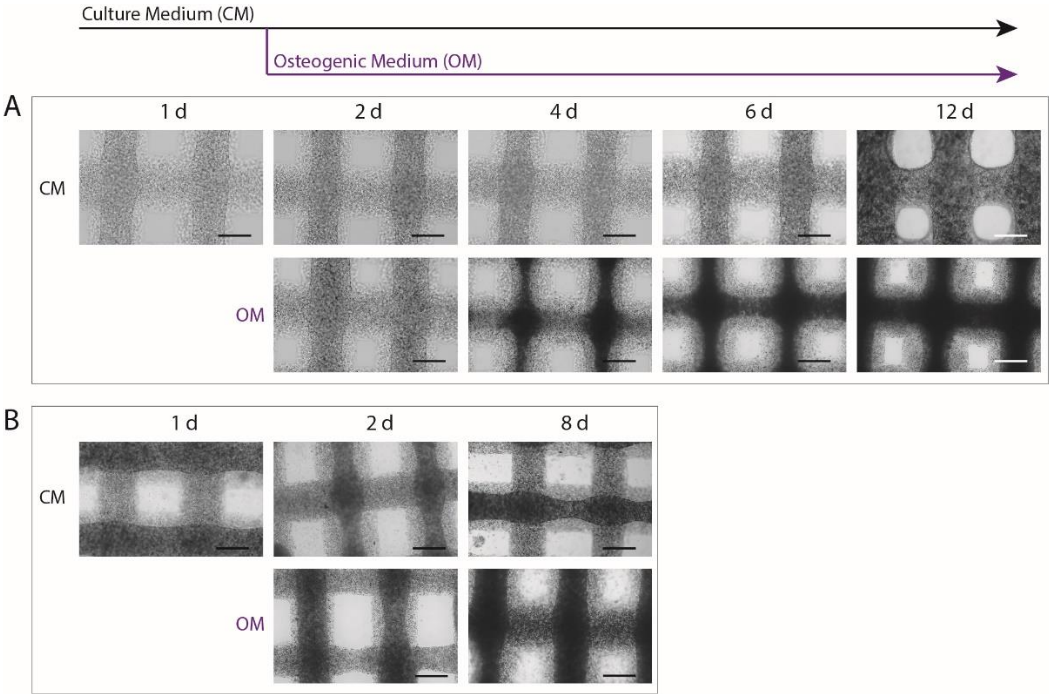

Figure S8. Representative microscopic images of printed lattice structure during culture using culture medium (CM) or osteogenic medium (OM), with cell concentrations of (A) 5×10^6 and (B) 1×10^8 Saos-2 cells mL^{-1} in matrix (7.5 wt% GelMA). These figures in (A) and (B) are

obtained from the same experiments as in Figure 5D and 5I, respectively, but shown in different magnifications. Scale bars: 500 μm.

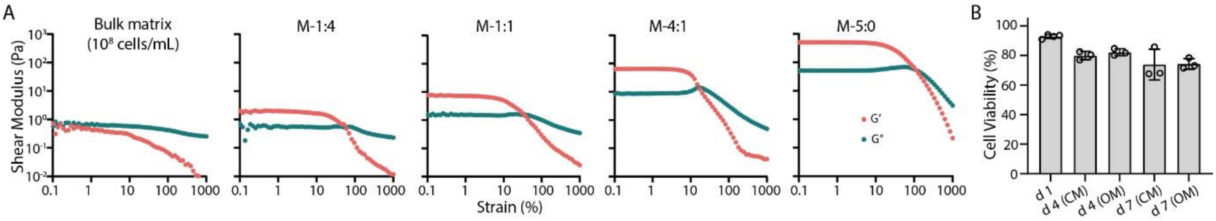

Figure S9. (A) Strain sweeps of MTP bioinks at different component ratios with bulk matrix (containing 10^8 cells/mL) and bulk microgels (M-5:0) as controls. (B) Quantified cell viability in bioprinted constructs during culture based on M-1:1 bioink.

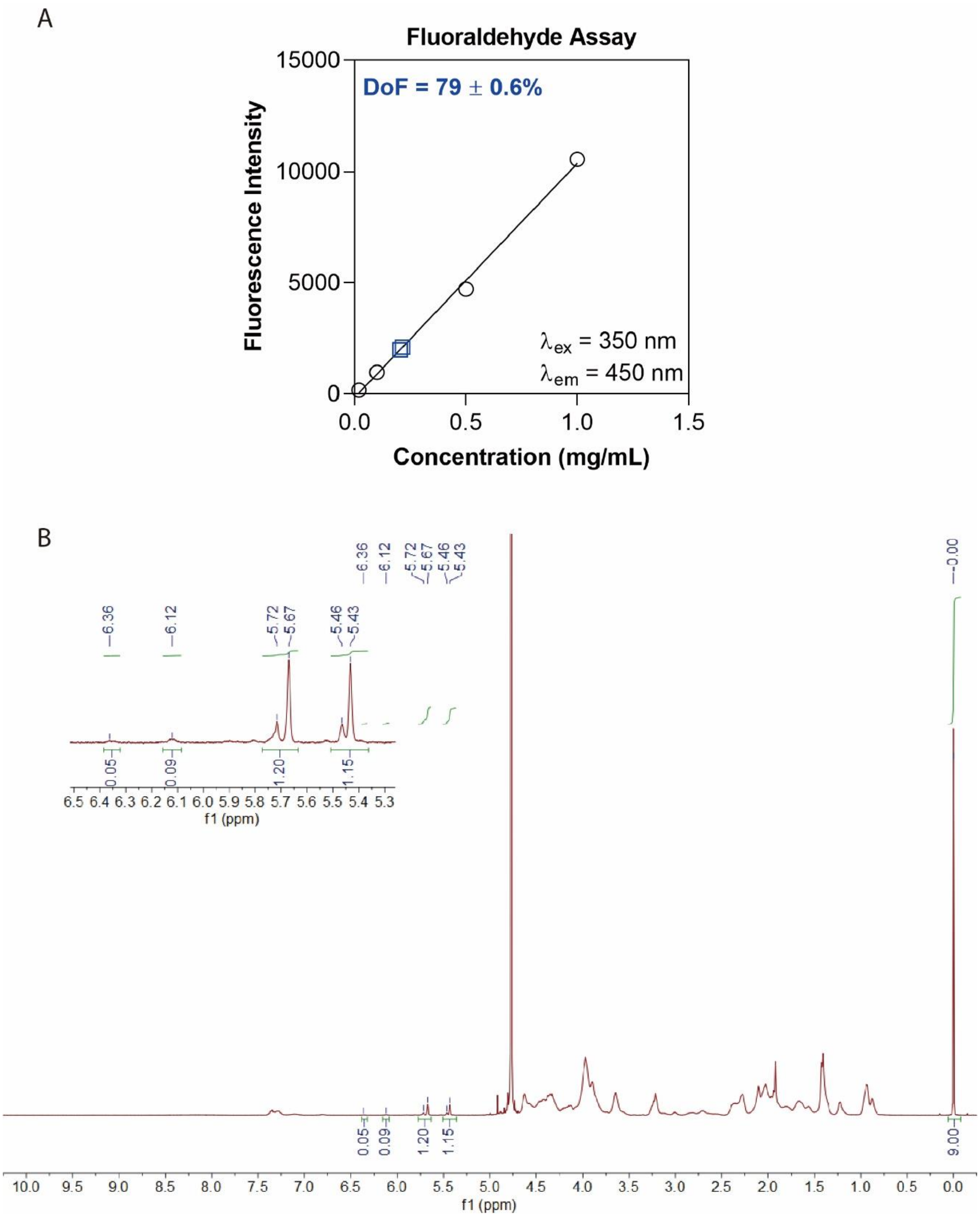

Figure S10. Characterization of fish GelMA. (A) Fluoraldehyde assay on Fish GelMA to determine the degree of functionalisation based on amine modification. Data show as mean ± S.D., n = 3. (B) Representative ^{1}H NMR spectra of F-GelMA with sodium 3-trimethylsilyl-propionate-2,2,3,3-d_4 (TMSP) as an internal standard.
